# The variation of FiO_2_ with circuit type and peak inspiratory flow rate during non-invasive respiratory support using domiciliary ventilators and its significance during the COVID-19 pandemic

**DOI:** 10.1177/1751143720980280

**Published:** 2022-05

**Authors:** Ben Messer, Hilary Tedd, Tom Doris, Andrew Mountain, Cris Gatilogo, Milind Sovani

**Affiliations:** 1 North East Assisted Ventilation Service, Royal Victoria Infirmary, Newcastle-upon-Tyne NHS Hospitals NHS Foundation Trust, Newcastle-upon-Tyne, UK; 2 Electronics and Medical Engineering, Royal Victoria Infirmary, Newcastle-upon-Tyne NHS Hospitals NHS Foundation Trust, Newcastle-upon-Tyne, UK; 3 Department of Respiratory Medicine, Nottingham University Hospitals NHS Trust, Nottingham, UK

**Keywords:** Ventilation, continuous positive airway pressure, non invasive ventilation, oxygen, FiO_2_, peak inspiratory flow rate, COVID-19

## Abstract

**Background:**

The COVID-19 pandemic has resulted in increased admissions with respiratory failure and there have been reports of oxygen failure and shortages of machines to deliver ventilation and Continuous Positive Airway Pressure (CPAP). Domiciliary ventilators which entrain room air have been widely used during the pandemic. Poor outcomes reported with non-invasive respiratory support using ventilators which lack an oxygen blender could be related to an unreliable Fraction of inspired O_2_ (FiO_2_). Additionally, with concerns about oxygen failure, the variety of ventilator circuits used as well as differing peak inspiratory flow rates (PIFR) could impact on the FiO_2_ delivered during therapy with domiciliary ventilators.

**Methods:**

In a series of bench tests, we tested the effect of choice of circuit and different PIFR on the FiO_2_ achieved during simulation of ventilation and CPAP therapy using domiciliary ventilators.

**Results:**

FiO_2_ was highly dependent upon the type of circuit used with circuits with an active exhalation valve achieving similar FiO_2_ at lower oxygen flow rates than circuits using an exhalation port. During CPAP therapy, high PIFR resulted in significantly lower FiO_2_ than low PIFR.

**Conclusions:**

This study has implications for oxygen usage as well as delivery of non-invasive respiratory support during therapy with domiciliary ventilators when these are used during the second wave of COVID-19.

## Introduction

The COVID-19 crisis caused by SARS-CoV-2 has resulted in significant increases in critical care utilisation across the world. Resource limitation has been a theoretical and actual problem with concerns about the availability of ventilators and oxygen supply. In the UK, the supply of ventilators has prompted an emergency response^1^ and there has been a reported incidence of oxygen failure within a UK hospital.^2^

Contingency planning has occurred and critical care units have used ventilators usually used in a home setting to deliver Non-Invasive Ventilation (NIV) and Continuous Positive Airway Pressure (CPAP) to patients in critical care.^3^ The use of CPAP in ward environments has also been reported and recommended^4^ and is the subject of a current randomised controlled trial (Recovery-RS Trial ISRCTN169120750). However, outcomes reported from CPAP therapy have been variable with mortality rates of 76% reported in one series.^5^

Breathing circuits for NIV and CPAP use a viral filter placed at the patient end of the breathing circuit to allow scavenging of SARS-CoV-2 and reduce transmission to staff and other patients. This has been the subject of national UK guidance.^6^

Ventilators which are used to deliver CPAP and NIV in a home setting have the disadvantage of separate administration of oxygen and entrainment of room air to meet peak inspiratory flows, rather than using an oxygen blender as critical care ventilators do.^7^ This results in uncertainty about the fraction of inspired oxygen (FiO_2_). Furthermore, room air is entrained from the environment and peak inspiratory flow rates (PIFR) are high during acute respiratory failure resulting in dilution of oxygen and lower FiO_2_.^8^ This may in part explain poor outcomes with CPAP therapy delivered by domiciliary ventilators.^5^ There have been several reports on social media platforms during the COVID-19 pandemic which have suggested that high FiO_2_ (up to 0.85) is achievable with non-invasive CPAP and NIV. However these experiments are often conducted whilst breathing comfortably. These have been replicated in bench studies using very low NIV pressures which result in low PIFR and therefore less dilution of inhaled oxygen with entrained air. For example, Schwartz and colleagues found an FiO_2_ of 0.78 with 10 L/min of oxygen administered and low NIV pressures of 10/5.^9^ It is important during experiments on CPAP to mirror as closely as possible the high PIFR seen during episodes of acute respiratory failure.

In breathing circuits there are three circuit configurations which allow exhalation without rebreathing carbon dioxide (CO_2_).

1. A vented mask where exhalation occurs via vents in the mask or at the connection between the mask and the ventilator tubing.
2. An exhalation port within the ventilator tubing.
3. An active exhalation valve within the ventilator tubing.

Vented masks, which are common in a home NIV and CPAP setting, are not recommended during the COVID-19 crisis due to inability to place a filter closer to the patient than the vents in the mask and therefore increasing the risk of environmental transmission of SARS-CoV-2.^6^

**Figure 1.**
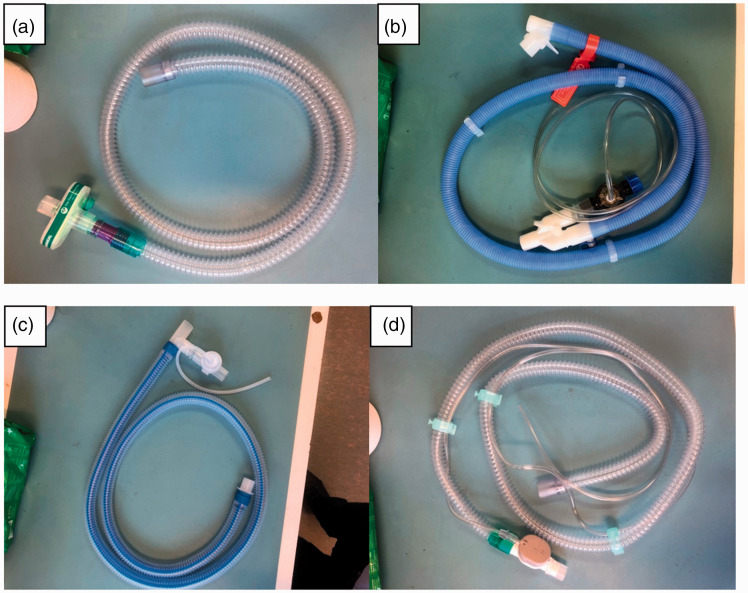
Circuits used. Single Limb Circuit with an Exhalation port (a). Active exhalation port in a dual limb circuit (b). Active exhalation port in a co-axial circuit (c). Active exhalation port in single limb circuit (d).

Most acute NIV is delivered using a single limb circuit with an exhalation port in the ventilator tubing which is the national recommendation for acute CPAP and NIV during COVID-19 in the UK (Figure 1, Circuit A).^6^ The use of a single limb circuit with an exhalation port has a significant disadvantage which is that in order to eliminate CO_2_, gas flow from the ventilator and therefore entrainment of room air continues during exhalation. This depends on the PEEP but is typically at least 20 L/min. This has the effect of diluting administered oxygen and potentially reducing FiO_2_.

**Figure 2.**
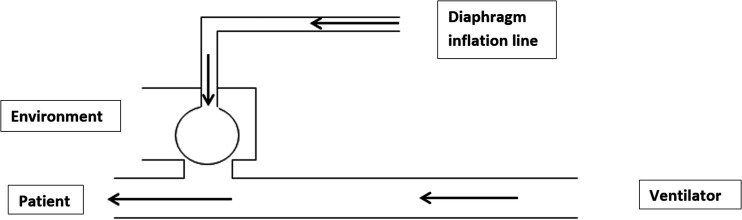
Active exhalation valve with arrows showing airflow during inspiration.

**Figure 3.**
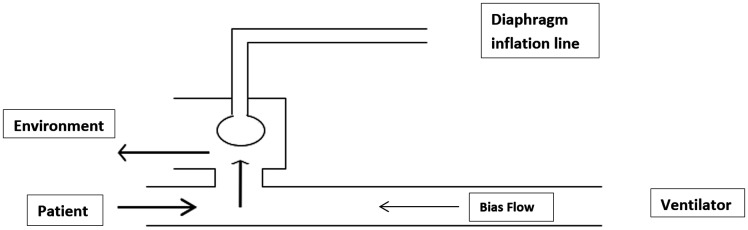
Active exhalation valve with arrows showing airflow during expiration.

An active exhalation valve is a large hole in the ventilator circuit which is blocked by a balloon which is inflated during inspiration but deflated during expiration to allow CO_2_ to be exhaled from the circuit. When an active exhalation valve is used, due to the large aperture (and therefore low resistance) of the hole via which exhalation occurs, flows during exhalation can be much lower and oxygen administered is consequently less diluted by entrained room air. This flow, called bias flow, is 8 L/min and serves to wash out CO_2_ from the circuit. There is therefore a theoretical reason for the FiO_2_ to be lower in a circuit with an exhalation port compared to a circuit with an active exhalation valve. A schematic diagram of the function of an active exhalation valve in inspiration and expiration is shown in Figures 2 and 3 respectively.

National shortage of equipment has extended to a shortage of these active exhalation valves (Breas personal communication). There are 3 types of these valves which can be used in different circuit configurations:

1. Dual limb circuit with separate inspiratory and expiratory limbs. (Figure 1, Circuit B).
2. Coaxial circuit where the inspiratory and expiratory limbs are either run in parallel or one within the other (Bain circuit). (Figure 1, Circuit C).
3. An active exhalation valve within a single limb circuit. (Figure 1, Circuit D).

Due to concerns about oxygen usage, ventilator availability and the well-reported use of home ventilators for acute NIV and CPAP, there is a requirement to define the FiO_2_ with different breathing circuit configurations during different modes of ventilation and also define the FiO_2_ achieved during CPAP whilst breathing at a high PIFR. This may result in recommendations which could reduce the amount of oxygen usage in the future, including during the second wave of COVID-19 admissions.

We conducted experiments on different modes of ventilation using different circuit configurations to model controlled ventilation via an endotracheal tube, to model NIV using pressure support ventilation via a facemask and CPAP via a facemask to investigate the FiO_2_ achieved.

## Methods

We conducted three experiments. Firstly, we assessed the effect of different circuit configurations and ventilator modes on FiO_2_ during mandatory ventilation (Experiment 1). Secondly, we assessed the effect of different circuit configurations and ventilator modes on FiO_2_ during supported ventilation which would model NIV (Experiment 2). Finally, we assessed the effect of altering PIFR on FiO_2_ during CPAP therapy (Experiment 3).

### Experiment 1

We used a Vivo 50 ventilator (Breas, Gothenburg, Sweden). Ventilatory modes and settings are detailed in Table 1. Mandatory ventilation (Pressure Control and Volume Control) was delivered into a test lung to simulate ventilation via an endotracheal tube or tracheostomy with settings approximating to standard clinical settings. Pressure Control ventilation was adjusted to achieve tidal volumes of 500mls. The test lung used was a Draeger 2 litre test lung with angled connector and 7 mm restrictor (Draeger Medical UK).

**Table 1.** Ventilatory settings.

Mode	Tidal Vol	Frequency	IPAP	EPAP	I:E Ratio
VCV	500	14	N/A	10	1:2
PCV	N/A	14	28	10	1:2
PS	N/A	N/A	25	10	N/A
CPAP	N/A	N/A	N/A	5–15	N/A

EPAP: expiratory positive airway pressure; IPAP: inspiratory positive airway pressure; PCV: pressure control ventilation; PS: pressure support; VCV: volume control ventilation.

### Experiment 2

We used a Vivo 50 ventilator (Breas, Gothenburg, Sweden). Ventilatory settings are detailed in Table 1. Pressure Support ventilation was delivered to a member of the team (BM) with a voluntary respiratory rate of approximately 20 breaths/min. The mask used was a Performatrak (Philips, Pennsylvania, USA) which is a non-vented mask.

For both experiment 1 and 2, the circuits used are detailed in Figure 1. All circuits contained a bacterial/viral filter (Intersurgical, Wokingham, UK) placed between the subject (test lung or member of the research team) and the exhalation port or valve.

(Figure 1, Circuit A) shows an exhalation port (Intersurgical)

(Figure 1, Circuit B) shows an active exhalation valve in dual limb circuit (Breas)

(Figure 1, Circuit C) shows an active exhalation valve with a co-axial circuit (Breas)

(Figure 1, Circuit D) shows an active exhalation valve in a single limb circuit (Intersurgical)

### Experiment 3

We used the following machines with CPAP capability:

- Vivo 50 Breas, Gothenburg, Sweden
- NIPPY 3+ Breas, Stratford-upon-Avon, UK
- AirSense 10, ResMed Ltd, NSW 2153, Australia

We used a single limb circuit with an exhalation port with the circuit configuration following national UK advice (Figure 1, Circuit A). An active exhalation valve cannot be used in CPAP mode. PIFR were voluntarily adjusted by the member of the team to give measured values of 60-70 L/min (comfortable breathing) and 110-130 L/min (rapid breathing).

For all experiments, oxygen flow rates were adjusted in 5 L/min increments from 5-15 L/min. Flows were measured by a VT plus HF gas flow analyser (Fluke Biomedical, Washington, USA). FiO_2_ was measured using a side-stream gas analyser (SAM module, GE Healthcare, USA) placed between the exhalation port or active exhalation valve and the subject and attached to a Dash 4000 Monitor (GE Healthcare, USA).

## Results

### Experiment 1

**Figure 4.**
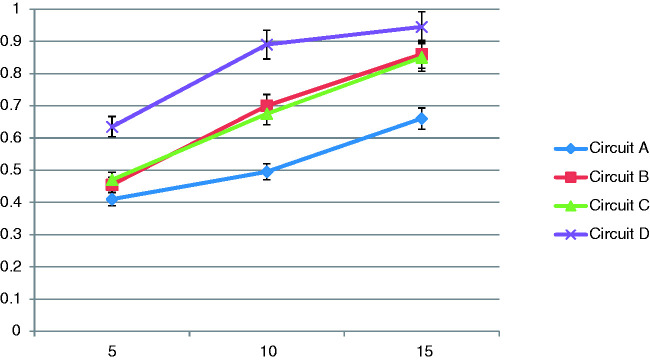
Variation of FiO_2_ with circuit type and oxygen flow rates with Volume Control Ventilation.

**Figure 5.**
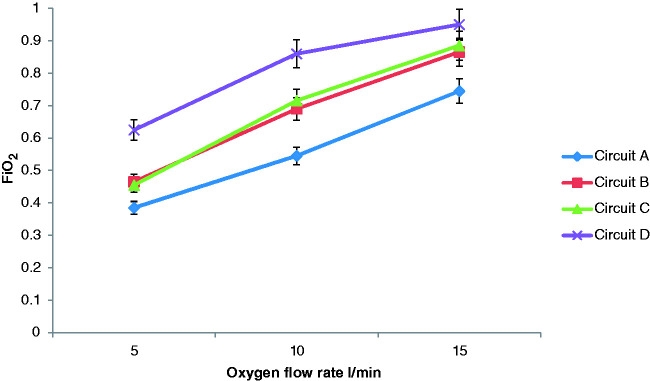
Variation of FiO_2_ with circuit type and oxygen flow rates with Pressure Control Ventilation.

During mandatory ventilation, the variation of FiO_2_ with mode, circuit configuration and oxygen flow rates is shown in Table 2. The variation of FiO_2_ with circuit configuration and oxygen flow rates for Volume Control Ventilation (VCV) and Pressure Control Ventilation (PCV) are shown graphically in Figures 4 and 5 respectively.

**Table 2.** FiO_2_ with different modes, circuits and Oxygen flow rates.

Mode	O2 flow (L/min)	Single limb	Active exhalation valve in dual limb circuit	Active exhalation valve in coaxial circuit	Active exhalation valve in single limb circuit
VCV	5	0.39–0.43	0.43–0.48	0.45–0.49	0.62–0.65
VCV	10	0.47–0.52	0.68–0.72	0.66–0.69	0.88–0.90
VCV	15	0.60–0.72	0.83–0.89	0.84–0.86	0.93–0.96
PCV	5	0.34–0.43	0.44–0.49	0.44–0.47	0.61–0.64
PCV	10	0.47–0.62	0.67–0.71	0.69–0.74	0.85–0.87
PCV	15	0.71–0.78	0.86–0.87	0.87–0.90	0.94–0.96
PS	5	0.24–0.25	0.36–0.37	0.25–0.32	0.31–0.32
PS	10	0.31–0.33	0.42–0.43	0.38–0.39	0.37–0.38
PS	15	0.38–0.39	0.48–0.50	0.46–0.47	0.48–0.49

FiO_2_ varies with the circuit used. A higher FiO_2_ was seen in any circuit with an active exhalation valve compared to a single limb circuit with an exhalation port during mandatory ventilation in either volume control or pressure control ventilation. An FiO_2_ of greater than 0.83 was seen at an oxygen flow rate of 15 L/min in all circuits using active exhalation valves during mandatory ventilation. This was particularly noted with the active exhalation valve in the single limb circuit in which an FiO_2_ of greater than 0.85 was seen during mandatory ventilation with an oxygen flow rate of 10 litres/minute. The single limb circuit with an exhalation port resulted in a similar FiO_2_ at oxygen flows of 10 and 15 L/min during mandatory ventilation as circuits with active exhalation valves with oxygen flows of 5 and 10 L/min respectively.

### Experiment 2

**Figure 6.**
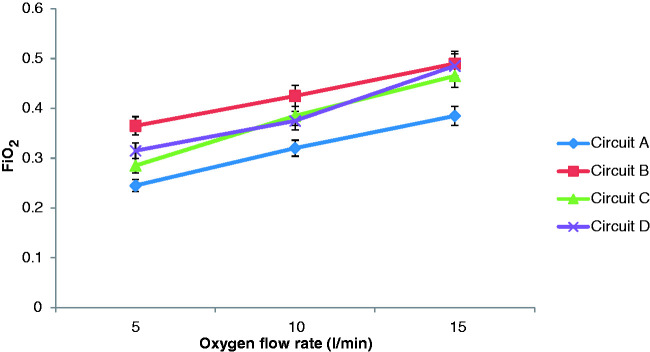
Variation of FiO_2_ with circuit type and oxygen flow rates with Pressure Support Ventilation.

During pressure support ventilation, the variation of FiO_2_ with circuit configuration and oxygen flow rates is shown in Table 2. The variation of FiO_2_ with circuit configuration and oxygen flow rates for Pressure Support (PS) is shown graphically in Figure 6.

During pressure support ventilation, the FiO_2_ achieved was much lower than with the mandatory ventilation. The maximum FiO_2_ achieved was 0.5 at oxygen flows of 15 L/min. Again, FiO_2_ achieved in a circuit with an active exhalation valve was higher than that in a single limb circuit with an exhalation port. The single limb circuit with an exhalation port resulted in a similar FiO_2_ at oxygen flows of 10 and 15 L/min during supported ventilation as circuits with an active exhalation valve with oxygen flows of 5 and 10 L/min respectively.

### Experiment 3

**Figure 7.**
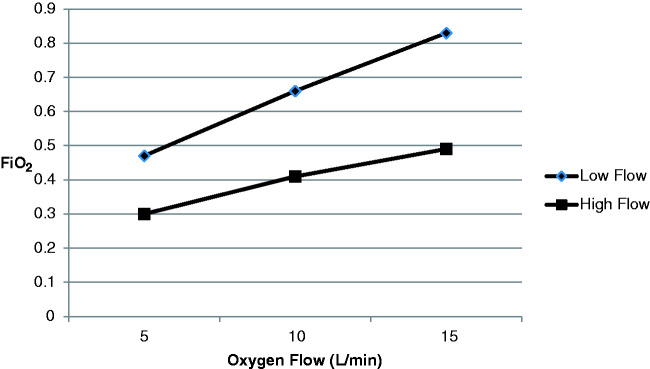
Average FiO_2_ results from all CPAP machines at high and low PIF rates with CPAP 10cmH_2_O.

During CPAP the variation of FiO_2_ with PIFR and oxygen flow rates is shown in Table 3 and graphically in Figure 7.

**Table 3.** FiO_2_ with low (60–70 L/min) and high (110–130 L/min) PIFR, CPAP settings and oxygen flow rates.

Oxygen flow	5 L/min	10 L/min	15 L/min
CPAP 5 (Low PIFR)	0.54	0.72	0.87
CPAP 5 (High PIFR)	0.30	0.37	0.44
CPAP 10 (Low PIFR)	0.47	0.66	0.80
CPAP 10 (High PIFR)	0.30	0.40	0.45
CPAP 15 (Low PIFR)	0.43	0.61	0.78
CPAP 15 (High PIFR)	0.27	0.35	0.39
Mean (Low PIFR)	0.48	0.66	0.82
Mean (High PIFR)	0.29	0.37	0.43

Data are presented in Table 3 as mean FiO_2_ of all CPAP machines as results were similar between machines.

Data are presented in Figure 7 as means of all three CPAP machines and at a CPAP of 10cmH_2_O as this figure corresponds to median CPAP values previously reported.^4^

During CPAP administration FiO_2_ was highly dependent upon PIFR. Mean FiO_2_ results whilst administering 15 L/min of oxygen averaged across all machines and at all CPAP values were 0.82 for low PIFR and 0.43 for high PIFR (Table 3). Similar differences between high and low PIFR were seen at oxygen flow rates of 5 and 10 L/min with the FiO_2_ achieved with high PIFR just over half of that at low PIFR.

FiO_2_ increased in all experiements and circuits with an increase in oxygen flow rates.

## Discussion

We have shown that the FiO_2_ varies with the type of circuit used, the mode of respiratory support and the oxygen flow rate. We have also shown that during spontaneous breathing with CPAP there is a large difference in FiO_2_ when breathing comfortably at low PIFR compared to rapid breathing with high PIFR which may more closely resemble the breathing pattern of patients who present with acute respiratory failure. We will discuss the individual findings below.

### Variation of FiO_2_ with circuit

During mandatory or supported ventilation with a single limb circuit and an exhalation port, a lower FiO_2_ was seen than with any of the circuits which used an active exhalation valve. When using a single limb circuit with an exhalation port, the aperture of the port needs to be small to ensure adequate ventilation during inspiration. This makes complete exhalation from the exhalation port impossible without continued flow from the ventilator during expiration. It is for this reason that Expiratory Positive Airway Pressure (EPAP) on these ventilators cannot be set below 3-4cmH_2_O. The constant flow during expiration offers a degree of EPAP. Flow rates of at least 20 L/min are typical. This has the effect of diluting the oxygen added to the circuit during expiration and therefore reducing the FiO_2_. The same is not true of circuits where exhalation occurs via an active exhalation valve. Here, there is a balloon which inflates to block a hole in the circuit during inspiration to ensure that all the gas is used to ventilate the patient. This balloon then deflates during expiration to open the hole and allow exhalation via the hole which is of large aperture and therefore offers minimal resistance to exhalation. This allows flow from the ventilator to reduce during expiration to bias flow (8 L/min) and therefore the remaining gas in the ventilator circuit is less diluted with entrained room air (Figures 2 and 3).

### Variation of FiO_2_ with mode of ventilation

A higher FiO_2_ was seen with mandatory ventilation than with pressure support or with CPAP. During mandatory ventilation, the flows into the patient are controlled. We used a frequency of 14bpm so that each breath lasted 4.3 seconds. A third (1.4 seconds) of this breath was spent during inspiration. With a tidal volume of 500mls this gives a flow of (500 ÷ 1.4) 349 ml/s. Multiplying this by 60 gives a flow rate of 20.9 L/min. Flow during expiration is less which is why FiO_2_ approached 1.0 with mandatory ventilation and 15 L/min oxygen administered. The situation during pressure control ventilation is more complex since the inspiratory flow rapidly reaches a peak and then reduces later during the inspiratory cycle however the mean flow during inspiration remains the same assuming that the pressures are set to give the same tidal volume as volume control ventilation and the inspiratory time is the same.

During pressure support ventilation, the flow is dependent upon the level of pressure support, the resistance of the lung and patient effort which increases spontaneously driven flows in the circuit. During pressure support, peak inspiratory flows of well in excess of 100 L/min are common and are likely to be even higher in patients with acute respiratory failure. This has the effect of increasing the contribution of entrained room air to the gas flowing into the patient and therefore reducing the FiO_2_ obtained.

### Variation of FiO_2_ with oxygen flow rates

This is perhaps the most expected finding of these experiments. Using the example above of a flow rate during inspiration of just over 20 L/min, a set flow rate of 5,10 and 15 L/min of oxygen represents ¼, ½ and ¾ of the overall flow into the patient, the rest being made up of entrained room air.

### Variation of FiO_2_ with varying flow rates during CPAP

Again, varying the PIFR will alter the FiO_2_ due to the requirement to increase entrained room air at high PIFR. During comfortable breathing at about 60 L/min and with oxygen flow rates of 15 L/min, 45 L/min of entrained room air are required during peak inspiration, representing ¾ of all flow during this time. With peak inspiratory flows of 120 L/min, the entrained room air is now 7/8 of the overall flow during peak inspiratory flows which dilutes the administered oxygen.

Our experiments have a number of implications in the setting of resource limitation (such as the COVID-19 crisis) where home ventilators are used to deliver invasive ventilation, acute NIV or CPAP. Firstly, on the basis of oxygen utilisation, we can recommend that for mandatory ventilation, including ventilation via an endotracheal tube or tracheostomy, an active exhalation valve should be used as opposed to a single limb circuit with an exhalation port. The recommendation remains when the patient begins to wean from ventilatory support and begins to breathe with pressure support. The recommendation also remains for patients on NIV. The circuit therefore does not require further modification during an individual patient use. This is the first study to our knowledge which has investigated the effect of the circuit used on FiO_2_ during mandatory and supported ventilation.

We used three different types of active exhalation valves, which gives clinicians a choice of suppliers during times when respiratory equipment can be difficult to source.

Since an equivalent FiO_2_ can be achieved with 5 and 10 L/min using an active exhalation valve compared to 10 and 15 L/min respectively using a single limb circuit with an exhalation port, a reduction in oxygen utilisation of up to 50% can be achieved. This may have significant beneficial effects during the second wave of the COVID-19 pandemic and for future situations which may arise which risk overwhelming current critical care resources.

We have also shown that FiO_2_ depends heavily on PIFR during CPAP. Clinicians should exercise caution when interpreting experiments on FiO_2_ which are conducted during comfortable breathing, as this may not simulate the conditions experienced during an admission with acute respiratory failure. Such experiments should not be used to estimate FiO_2_ or to help calculate the potential oxygen requirement of a hospital ward during pandemics and other times when increases in non-invasive respiratory support is required.

Where available, ventilators with an oxygen blender should be recommended to be certain of the FiO_2_ delivered.

There are some limitations of our study. Firstly, we did not investigate the effect of the site of entrainment of oxygen into the circuit on FiO_2_. This is an important omission; however we set the circuit up using UK national recommendations during the COVID-19 pandemic.^6^ In particular, we did not administer oxygen directly into the mask as this would risk spread of SARS-CoV-2 should the tubing used to administer oxygen become disconnected. Studies which have previously investigated the site of oxygen administration have found conflicting results.^9^,^10^ Where there was a difference in FiO_2_ according to the site of oxygen administration, the size of this effect was small.

Secondly, we have assumed that during an episode of acute respiratory failure, the PIFR is double that during comfortable breathing. An important further experiment would be to quantify this with a patient series to investigate the PIFR in patients during an episode of acute respiratory failure. Most modern ventilators used to deliver acute CPAP will measure the peak inspiratory flow.

Finally, most acute respiratory support in a non-critical care setting is delivered using single limb circuits with an exhalation port. Changing the circuit will have implications for staff education and risk management which individual institutions and clinicians will need to consider prior to introducing any change to normal practice.

## Conclusion

A breathing circuit with an active exhalation valve offers a potential reduction in oxygen utilisation of up to 50% during both mandatory and supported ventilation, which has important implications during times of resource limitation such as during the COVID-19 pandemic.

FiO_2_ is dependent upon PIFR during CPAP therapy and caution should be exercised when interpreting the FiO_2_ calculated during experiments conducted whilst breathing comfortably.
